# Stroke Neuroprotection: Targeting Mitochondria

**DOI:** 10.3390/brainsci3020540

**Published:** 2013-04-19

**Authors:** Lora Talley Watts, Reginald Lloyd, Richard Justin Garling, Timothy Duong

**Affiliations:** 1 Department of Cellular and Structural Biology, University of Texas Health Science Center San Antonio, San Antonio, TX 78229, USA; 2 Department of Neurology, University of Texas Health Science Center San Antonio, San Antonio, TX 78229, USA; 3 Research Imaging Institute, University of Texas Health Science Center San Antonio, 7703 Floyd Curl Drive, San Antonio, TX 78229, USA; E-Mails: lloydr@uthscsa.edu (R.L.); duongt@uthscsa.edu (T.D.); 4 School of Medicine, University of Texas Health Science Center San Antonio, 7703 Floyd Curl Drive, San Antonio, TX 78229, USA; E-Mail: garling@livemail.uthscsa.edu

**Keywords:** stroke, purinergic receptor, methylene blue, mitochondria, neuroprotection, superoxide dismutase

## Abstract

Stroke is the fourth leading cause of death and the leading cause of long-term disability in the United States. Blood flow deficit results in an expanding infarct core with a time-sensitive peri-infarct penumbra that is considered salvageable and is the primary target for treatment strategies. The only current FDA-approved drug for treating ischemic stroke is recombinant tissue plasminogen activator (rt-PA). However, this treatment is limited to within 4.5 h of stroke onset in a small subset of patients. The goal of this review is to focus on mitochondrial-dependent therapeutic agents that could provide neuroprotection following stroke. Dysfunctional mitochondria are linked to neurodegeneration in many disease processes including stroke. The mechanisms reviewed include: (1) increasing ATP production by purinergic receptor stimulation, (2) decreasing the production of ROS by superoxide dismutase, or (3) increasing antioxidant defenses by methylene blue, and their benefits in providing neuroprotection following a stroke.

## 1. Stroke: A Brief Overview

Stroke is an increasingly prevalent clinical condition, especially with a gradually aging population. Nearly 800,000 strokes occur in the United States each year, ranking it as the fourth leading cause of death behind heart disease, cancer and chronic lower respiratory disease [1]. A stroke is clinically defined as the sudden loss of oxygen to brain tissue in a localized area due to inadequate blood flow. The American Heart Association (AHA) classifies stroke into three categories: ischemic (clots), hemorrhagic (bleed), and transient ischemic attack (TIA: mini stroke). The majority of reported strokes are ischemic strokes accounting for approximately 85% of all strokes, while non-traumatic hemorrhage account for up to 15% [2].

Ischemic stroke results from the blockade of an artery to the brain from in situ thrombosis or an embolus from another artery or the heart. Ischemic stroke results in macrophage infiltration, blood brain barrier breakdown and cellular dysregulation and infarction, followed by formation of edema [3,4,5,6]. In later stages, neurodegeneration and gliosis occur. Hemorrhagic stroke occurs due to a weakened blood vessel rupturing due to aneurysm or arteriovenous malformations, which results in bleeding into the surrounding brain tissue and subsequent tissue compression. TIA, as the name suggests, is a stroke caused by transient blockage usually less than 24 h (typically <5 min) of a vessel, and is often considered a warning stroke (by the AHA). TIAs are typically not associated with damage due to the short duration of the blockage. Blood flow deficit inhibits the delivery of oxygen, glucose and other nutrients from the blood, resulting in an expanding infarct core with a time-sensitive, salvageable peri-infarct penumbra that is the primary target for treatment strategies [7,8,9]. Penumbral tissue is potentially salvageable because this region still exhibits partial blood flow, oxygenation and metabolic activity [10]. Cellular integrity and function are still preserved to varying degrees within this area [11]. Due to the brain’s limited capacity to store glucose and the limited ability to utilize anaerobic metabolism, rapid neurodegeneration ensues. The amount of damage to the brain is determined by a number of factors including the location and duration of blockage, state of the circulatory system, collateral flow within the affected region, and the presence of other disease states [12,13,14]. A primary goal of developing therapeutic interventions in acute ischemic stroke is to preserve the ischemic, but still viable, cerebral tissue.

## 2. Pathological Mechanisms Associated with Stroke

**Figure 1:**
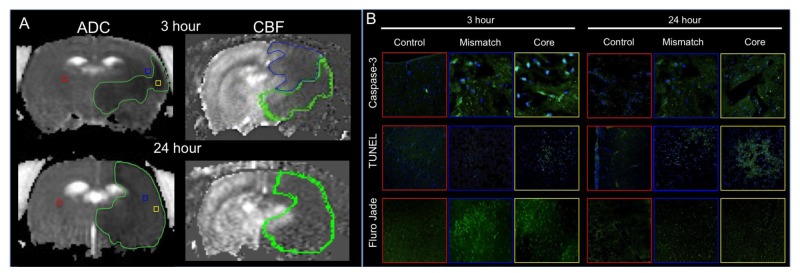
(A) Representative Apparent Diffusion Coefficient and Cerebral Blood Flow maps 3 and 24 h post-Middle Cerebral Artery Occlusion. Regions of interest are outlined on the cerebral blood flow map as core (green) and mismatch (blue) with the remaining regions considered healthy. (B) Within the selected regions of interest images were collected within the three hour defined regions as core, penumbra and mismatch and are represented by the square boxes of caspase-3, Terminal deoxynucleotidyl transferase dUTP nick end labeling (TUNEL) and Fluro Jade immunostaining. The data demonstrate an increase in caspase-3 expression within the core and penumbra areas compared to healthy tissue and a increase in expression of cell death markers TUNEL and Fluro Jade staining compared to control in the three hour post MCAO animals. By 24 h post-MCAO there is still an increase in caspase, TUNEL and Fluro Jade staining compared to control, however we see less Fluro Jade staining at 24 h compared to the 3 h animals suggesting a loss of tissue.

Impaired circulation within the brain is a complex multi-causal process. There are multiple mechanisms of injury at play following a stroke and changes as the lesion progresses. Some of these mechanisms include: glutamate excitotoxicity, activation of destructive enzymes (lipases, endonucleases, and proteases), inflammation, ion homoeostasis failure, and apoptosis [15,16,17,18,19,20,21,22,23,24]. The occlusion of a blood vessel causes rapid neuronal death in the area immediately surrounding the occluded vessel and is considered the core where irreversible damage occurs. The area immediately surrounding the core is considered the penumbra, where collateral blood flow can maintain cellular function by providing glucose and oxygen and is considered the salvageable tissue zone. Within the penumbra, neurons are functionally impaired but can remain viable for an extended period of time compared to the core, but will succumb to the injury if blood flow is not replenished resulting in an increasing lesion volume as shown in Figure 1A. The activation of the apoptotic cascade begins rapidly (within hours) and culminates in progressive cell death from the ischemic core towards the penumbra (Figure 1B). Therefore, many therapeutic strategies aim at preventing the cells within the penumbra from dying, thereby decreasing the overall lesion volume.

## 3. Current Therapeutic Approaches for Treatment of Stroke

The central nervous system (CNS) is particularly vulnerable to insults that result in cell death or damage, in part, because cells of the CNS have limited capacity for repair. As a result, stroke can result in debilitating and largely irreversible degradation of a patient’s cognitive and sensorimotor functions. The reestablishment of blood flow is imperative to minimize the damage caused by a stroke. Although there has been remarkable progress in our understanding of the pathophysiology of ischemic stroke, the only approved acute treatment is the use of recombinant tissue plasminogen activators (rt-PA) (Alteplase). Tissue plasminogen activator is a serine protease that is involved in the breakdown of blood clots by catalyzing the conversion of plasminogen to plasmin. However, rt-PA must be given within 4.5 h of symptom onset due to the high risk of hemorrhagic transformation, a restriction that results in only 3%–8% of all patients eligible to receive this therapy [25,26,27]. Additionally, thrombolytics can cause intracranial hemorrhage, which often results in death [28,29,30]. Therefore, there is an urgent need to find new therapies to extend the treatment window for patients suffering from ischemic stroke.

## 4. Mitochondrial Dysfunction in Stroke

Mitochondria have the primary function of providing cellular chemical energy in the form of ATP by oxidative phosphorylation via the electron transport chain, and as such they have been termed the cells “powerhouse”. Glucose is a major energy substrate for cells of the CNS (astrocytes and neurons) under physiological conditions [31,32,33]. Glucose taken up from the blood is metabolized through two major pathways: glycolysis and oxidative phosphorylation. Glycolysis, which occurs in cytosol, is the metabolism of glucose to pyruvate and lactate, and has a low-energy yield. In contrast, oxidative phosphorylation is the major pathway of ATP synthesis. It is driven by energy derived from electron transport in the mitochondria and is responsible for ~92% of total ATP production. In addition to ATP production, mitochondria are also involved in the regulation of cellular metabolism, calcium homeostasis, oxidative phosphorylation, generation of reactive oxygen species (ROS) and apoptosis (programmed cell death) (reviewed in [34]). 

ROS is a collective term used to describe a number of species that contain one or more unpaired electrons. Some of these species produced within a cell include superoxide, hydrogen peroxide, peroxyls, and hydroxyl radicals, among others. Under normal conditions the production of ROS is held in check primarily by antioxidant enzymes, however when a cell becomes stressed the production of ROS can exceed the antioxidant capacity and result in oxidative stress-related cell death. Oxidative stress is defined as an imbalance between the production of ROS and antioxidant defenses [35]. Mitochondrial respiratory chain complexes I and complex III are considered to be the major sites of cellular superoxide production [36,37,38] as ROS are produced as a byproduct of normal cellular metabolism of oxygen. The ROS produced during normal mitochondrial respiration are considered to be an important source for oxidative damage [39,40]. Cellular stress increases the level of ROS produced and can overwhelm the cells natural defenses resulting in increased oxidative stress and subsequent activation of cell death pathways. Mitochondrial dysfunction clearly plays a key role in a variety of forms of cell death, including ischemia [41,42], excitotoxic neurodegeneration [43], oxidant-induced stress [44] and apoptosis [45,46,47,48]. 

Mitochondrial destabilization resulting in dysfunctional mitochondria causes the activation of cell death pathways. This process is primarily thought to occur through mitochondrial membrane permeabilization. The permeabilization of the mitochondrial membrane allows for the release of mitochondrial proteins into the cytosol causing the initiation of caspase-dependent (through cytochrome *c* release) and -independent (through calpain- or poly(ADP-ribose) polymerase-1 mediated apoptosis inducing factor release) apoptotic pathways. For example, cytochrome *c* release induces the caspase cascade resulting in damage to proteins and DNA culminating in cell death. There have been a number of studies targeting these pathways; however many of these studies have only examined acute time periods (*i.e.*, assessment only up to one week post infarct). This limited time window of neuroprotection has yet to be established, but may be due to increased oxidative stress or other mitochondrial-independent mechanisms. 

It is well known that oxidative stress is a leading cause of oxidative damage in ischemic brain injuries such as stroke. This is especially true during reperfusion after cerebral ischemia. Increased levels of ROS have also been documented in aged neuronal cells [49,50,51]. The rate of oxidative metabolism in penumbral tissue has been suggested as the best predictor for eventual survival after stroke [52]. Cells have developed strong anti-oxidant defenses to scavenge ROS and minimize their destructive nature. There are numerous cellular mechanisms that provide protection from the damaging nature of ROS. These include superoxide dismutases (SODs), glutathione, vitamin A, C and E, catalase and other peroxidases.

ROS can induce apoptosis by depleting glutathione (GSH) or by changing cellular redox potentials [53]. Decreased GSH have also been observed in a number of senescent organisms including humans [54]. However, little is known about the cumulative effects of damage on astrocytes, whose primary function is to protect and support neuronal activity. It seems likely that a degradation of their supportive and neuroprotective functions would by itself contribute to cellular damage [55,56].

Over the last fifty years, there has been an explosion in the literature of multiple pathways associated with neurodegeneration with respect to disease states such as stroke, traumatic brain injury, ALS, Parkinson’s and Alzheimer’s disease, among others. It is becoming increasing clear that there is no single mechanism that underlies any pathology. However, we can predict that a majority of these pathways are linked to energy production, either directly or indirectly. Therefore, targeting one or more of three pathways may provide protection from ischemia by (1) increasing ATP production, (2) decreasing the production of ROS, and/or (3) increasing antioxidant defenses and will likely benefit key pathways involved in neuroprotection. The goal of this review is to focus on mitochondrial-dependent therapeutic agents that could target each of these mechanisms to provide neuroprotection following stroke. 

## 5. Increasing ATP Production with Purinergic Receptor Stimulation

As mentioned in an earlier section, focal cerebral ischemia manifests as a non-salvageable ischemic core immediately surrounding the blocked artery, caused by no blood flow, with an intermediate salvageable area, the penumbra, characterized by reduced blood flow. Initially, the neurons in the penumbra maintain their integrity by maintaining ATP levels near normal. However, if perfusion is not returned, these neurons lose their ability to generate ATP [57,58,59]. One mechanism to maintain cellular ATP levels is through the stimulation of exogenous purinergic receptors. Purinergic receptors are membrane-bound receptors expressed on almost all mammalian cells and are classified as either P2X-receptors (ligand-gated ion channels) or P2Y-receptors (G-protein-coupled receptors) [60,61]. 

Cellular damage induces a rapid efflux of ATP into the extracellular space, and may provide a mechanism to induce a cellular response to injury. Following hypoxia or focal ischemia, extracellular adenosine is rapidly accumulated and has been shown to reduce cerebral ischemic damage, supporting a role for purines as neuroprotective agents [62,63,64,65,66]. Ischemia, hypoxia and epilepsy also induce neuronal hyperexcitability and are thought to underlie associated neurodegeneration through excessive glutamate release and alterations in calcium homeostasis that can be mitigated by agonists of purinergic receptors [64]. Adenosine-based nucleotides mediate a wide variety of physiologic actions including regulation of platelet aggregation, muscle contraction, neurotransmission and epithelial cell communication and migration, many of which are mediated by metabotropic P2Y-receptors [67]. Metabotropic receptors are typically defined as receptors, when upon ligand binding activates a G-protein, which then activates a secondary messenger. The secondary messenger may then act to bind and open an ion channel or activate another intermediate molecule.

**Figure 2:**
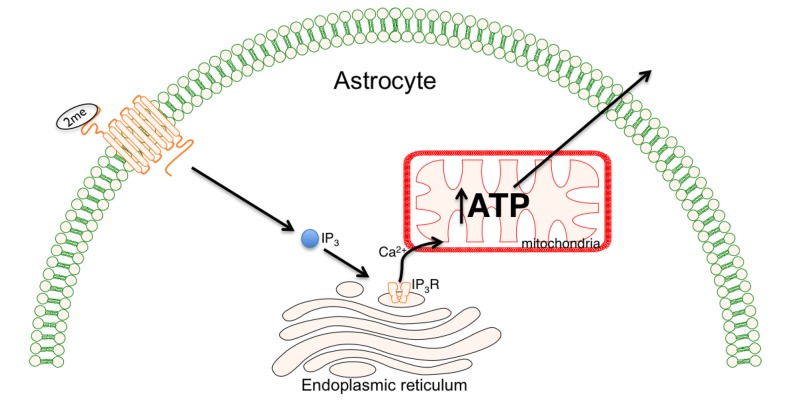
Neuroprotection can be enhanced by stimulating Calcium-dependent astrocyte mitochondrial metabolism. The binding of 2-methylthioadenosine diphosphate trisodium salt(2meSADP) to the purinergic receptor (P2Y_1_R) activates the Inositol triphosphate (IP_3_) cascade resulting in release of calcium (Ca^2+^) from endoplasmic stores. The result is increased adenosine triphosphate (ATP) production within the mitochondria which provides for increased cellular demands following injury.

P2Y-receptors consist of eight mammalian subtypes (P2Y_1,2,3,6,11,12,13,14_). A majority of the P2Y-receptors signal through coupling to Gq/11, with only the P2Y_12_, P2Y_13_ and P2Y_14_ signaling through Gi/o. Activation of P2Y-receptors initiates a number of signaling cascades including phospholipases (PLCβ, PLD, PLA2), adenylyl cyclase (AC) and mitogen-activated protein kinases (MAPK/MEK kinase). Purinergic receptor (P2Y_1_R) activation provides a mechanism whereby local extracellular signals can rapidly elevate intracellular calcium levels through increased production of inositol triphosphate (IP_3_) [68,69]. IP_3_-mediated calcium release increases mitochondrial calcium and consequently, increases respiration and ATP production [70,71,72,73] (Figure 2). 

**Figure 3:**
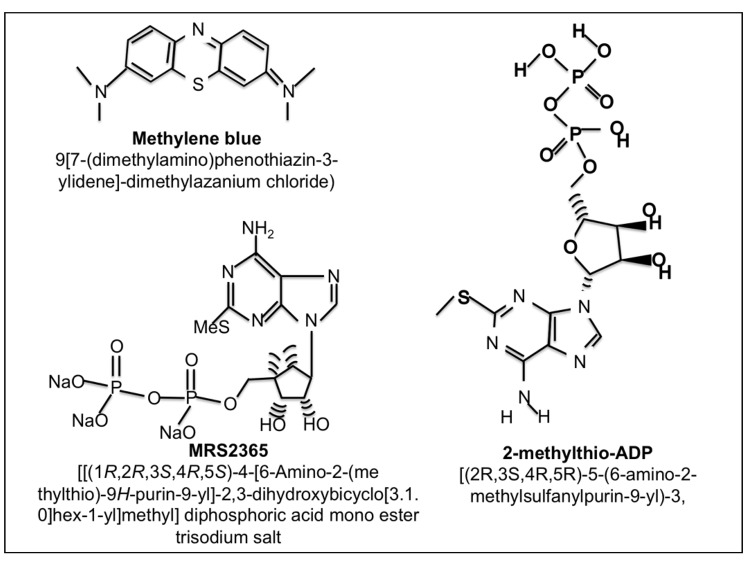
Chemical structures of Methylene Blue, 2-methylthio-ADP and MRS2365.

**Figure 4:**
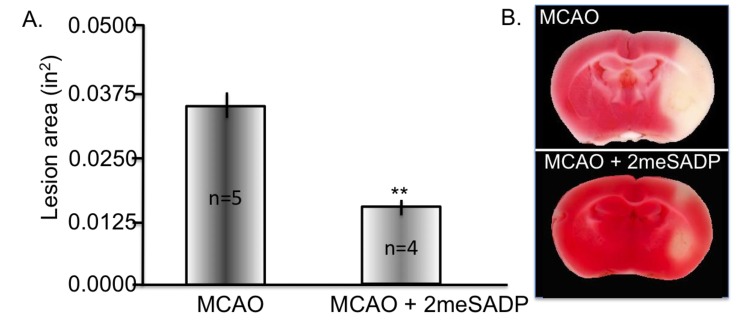
Purinergic receptor stimulation using 2-methylthioadenosine diphosphate trisodium salt (2meSADP) following middle cerebral artery occlusion (MCAO) rescues cortical cellular layers. Rats were treated with 100 μM 2meSADP 30 min post MCAO. Forty-eight hours following treatment the rats were sacrificed and triphenyltetrazolium chloride (TTC) staining was performed. (A) Histogram demonstrating the reduction in lesion volume with 2meSADP treatment. (B) Representative images from two different rats following MCAO with or without 2meSADP treatment. ** indicates *p* < 0.001 using student’s *t*-test.

2-Methylthioadenosine diphosphate trisodium salt (2meSADP) is a selective, potent purinergic agonist for the P2Y_1_, P2Y_12_ and P2Y_13_ receptors with EC_50_ values of 8.29 and 9.05, 19 nM, respectively [74]. The chemical structure is found in Figure 3. 2meSADP enhances mitochondrial metabolism and has been shown to have protective effects in hypotension, bleeding disorders, thromboembolism and cardiovascular disease [75,76,77,78]. Neuroprotection following a stroke can be enhanced by increasing astrocyte mitochondrial metabolism via P2Y_1_ receptor activation using the agonist 2meSADP or MRS 2365 [79,80]. Two studies utilized the Rose Bengal photothrombosis stroke model to determine the effect of 2meSADP [80,81]. In this model, the photosensitive dye, Rose Bengal, is administered by tail vein and excited through a thinned cranium with a 562 nm light using a confocal microscope. This model causes a permanent clot in the vessels exposed to the laser. Rose Bengal generates ROS, which activates tissue factor (TF), the initiator of the coagulation cascade, and consequently triggers an extrinsic coagulation cascade, therefore producing an ischemic lesion that is pathologically very relevant to clinical stroke [82]. 2meSADP was found to markedly reduced infarct size in this model in mice and was hypothesized to occur by increasing calcium sensitive mitochondrial metabolism in astrocytes through G-protein coupled purinergic receptor stimulation [83]. In addition, it was observed that cytotoxic edema formation was also reversed following 2meSADP administration suggesting the importance of maintaining mitochondrial metabolism in multiple facets of the pathological consequences of a stroke. More recently, these findings were extended to demonstrate that stimulation of P2Y_1_R with 2meSAP also enhances neuronal survival following a stroke using transgenic mice expressing yellow fluorescent protein in neurons. Following stoke the morphological changes associated with neuronal death such as beading and swelling of dendrites were assessed and found to be reversed with 2meSADP treatment [81]. Mitochondrial membrane potentials were also found to be diminished following an infarct and were repolarized upon 2meSADP administration [81]. Utilizing a cerebral ischemia/reperfusion model of stroke (MCAO with 60 min reperfusion) we assessed the ability of 2meSADP to reduce lesion volume. Rats treated with 2meSADP (200 µg dose) at the time of reperfusion had significantly reduced lesion volumes demonstrated by 2,3,5-triphenyltetrazolium chloride (TTC) staining (common method for visualizing stroke volume) forty-eight hours after stroke (Figure 4). In summary, 2meSADP treatment (1) reduces neuronal cell death; (2) reverses ischemia-induced brain damage and swelling; (3) correlates with increased mitochondrial metabolism and neuroprotection and (4) depends on the expression of the IP_3_ receptor on astrocytes.

Despite its apparent beneficial effects, a potential problem with 2meSADP treatment is its ability to induce platelet aggregation, and inhibit cAMP accumulation in platelets *in vitro* [84,85,86,87]. The ability of 2meSADP to induce platelet aggregation has been demonstrated to occur through the simultaneous activation of both P2Y_1_ and P2Y_12_ receptors, both of which are expressed on platelets. The activation of the P2Y_1_ receptor stimulates a G(alpha)q activation of phospholipase C causing hydrolysis of PtdIns(4,5)P2 to diacylglycerol (DAG) and IP_3_. DAG and IP_3_ then activate protein kinase C (PKC) increasing mobilization of calcium from intracellular stores. However, blocking the P2Y_12_ receptor prevents the aggregation of platelets [87,88]. While 2meSADP may induce platelet aggregation, there are additional agonists that specifically target the P2Y_1_ receptor such as MRS2365. MRS2365 is a highly selective P2Y1 receptor agonist with an EC_50_ value of 0.4 nM. MRS2365 has been demonstrated to have no activity at P2Y12 receptors and very low activity at P2Y13 receptors [89,90,91].

P2X receptors are ligand-gated ion channels that are considered nonselective cations channels responsible for excitatory postsynaptic responses. There are currently seven mammalian P2X receptor subtypes (P2X_1–7_). These receptors are widely distributed in both the central and peripheral nervous system and play key roles in regulation of renal blood flow, inflammatory responses and vascular endothelium physiology. The P2X receptors have most commonly been associated with mechanosensation, as well as chronic and neuropathic pain. The P2X_2_, P2X_4_ and P2X_6_ receptors are localized in many tissues however are most abundant in neurons. There is increasing evidence that P2X receptors may also play a role in modulation of cellular responses to injury such as ischemia. For example, it has been demonstrated that P2X_2_ and P2X_4_ receptors are elevated following ischemia, which are known to induce cell death pathways. However, non-specific antagonist treatment with sumarin prevented cell death following ischemic injury [92]. In addition, both the P2X_4_ and P2X_7_ receptors, typically found on microglia and also upregulated b oxygen glucose deprivation and may be involved in cortical damage. These results suggest that P2X receptor may also be involved in mechanisms underlying cell death following mitochondiral metabolism impairment. Additionally the use of antagonists may provide effective neuroprotective functions following conditions in which mitochondria is impaired.

In summary, the evidence suggests that following focal ischemia and hypoxia there is a rapid accumulation of extracellular ATP, suggesting an endogenous neuroprotective function of purines. Enhancing the concentration of ATP by purinergic stimulation may provide enhanced protection against cytotoxic edema formation and the induction of cell death pathways commonly associated with stroke. Unfortunately while the field of purniergic pharmacology has grown rapidly, efforts to develop therapeutics based on modulting these receptors for diseases of the CNS has been slow. We are optomistic that the development of novel ligands may aide in the potential to target the CNS following stroke and other neurodegerative disease states.

## 6. Decreasing Superoxide Production with Methylene Blue

Methylene Blue (MB), an FDA approved agent, has been used over the last 130 years for a number of applications ranging from textiles to, more recently, the treatment of neurodegenerative diseases in models of early stage Alzheimer’s disease and Parkinson’s disease [93]. MB, discovered and synthesized in the late 1800s by Heinrich Caro, is a cationic thiazine that contains a tri-heterocyclic thiazine ring structure (S(C_6_H_4_)_2_NH_4_) (see Figure 3 for structure) similar to many antipsychotic and antihistamine compounds; differing only by varying side chains [94]. The pharmacological use of methylene blue began in 1890s when Nobel laureate Paul Ehrlich discovered its usefulness in treating malaria. MB has since been used as a neuroprotective agent in drug-induced encephalopathy, dementia and manic-depressive psychosis [95,96,97] and been used to treat methemoglobinemia, and cyanide poisoning [98]. MB is believed to exert its neuroprotective effects through its pharmacological properties of being a potent antioxidant as well as a metabolic enhancer [98]. What makes MB an exciting intervention in neuro-related diseases is its rapid accumulation within the CNS, likely due to its high lipophilicity. MB has been shown to reach concentrations 10 times higher in the brain, compared to the circulation one hour after systemic administration [99]. It has recently been shown to reduce neurobehavioral impairment in animal models of Parkinson’s Disease [93] and cognitive decline in Alzheimer’s [100]. Furthermore, MB exhibits promising cardio- and neuroprotective properties in experimental cardiac arrest [101,102] and is effective in attenuating ischemia-reperfusion (I/R) syndrome [103] and increasing short-term survival after resuscitation from cardiac arrest [104]. 

**Figure 5:**
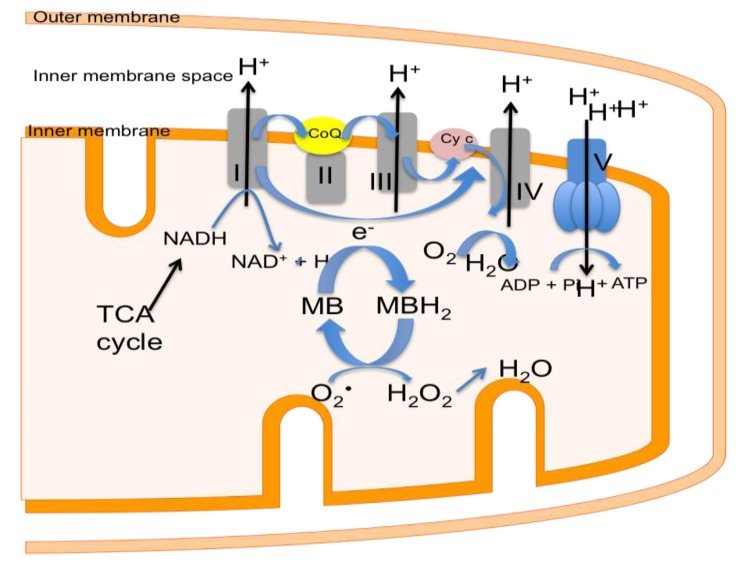
Within the inner mitochondrial membrane the oxidative phosphorylation pathway machinery is demonstrated (complex I–V) providing for the production of ATP. Methylene Blue (MB) provides an alternative mechanism of shuttling electrons by bypassing complex I–III, which allows for the continued production of ATP essential for cellular function while decreasing the number of ROS produced.

As described in a previous section, there is a small time frame after stroke to save the neurons residing in the penumbra that rely on ATP production for survival. Ordinarily, mitochondria produce both ATP and ROS. However, mitochondria have a limited ability to counteract ROS produced in the cell during oxidative phosphorylation. Stroke results in deficits in perfusion and thus increased production of ROS. The increased production of ROS in turn inundates the innate ability of mitochondria to counteract ROS, which leads to neuronal death. MB has been demonstrated to improve mitochondrial function and acts as an alternative electron carrier by shuttling electrons between NADH and cytochrome *c* which allows a mechanism for by passing complex I and III inhibition and reducing electron leakage (Figure 5). This in turn decreases ROS production thus decreasing oxidative damage. Wen *et al.* [105] demonstrated that *in vitro* in neuronal cultures, MB improves mitochondrial function. Specifically, MB was found to increase oxygen consumption rate even in the presence of inhibitors of complex I (rotenone), III (antimycin A) and V (oligomycin). In addition, ATP production was increased in neurons with MB treatment. Wen *et al.* [105] also found MB to be protective in a transient cerebral ischemia model using the intraluminal filament MCAO model in rats. Mitochondrial failure was observed as reduced activity of complex I, III and IV, 24 h post cerebral ischemia/reperfusion injury. This decrease in activities of complexes I, II and IV was attenuated with 500 µg/kg dose of MB and was hypothesized to be caused by bypassing complex I and III blockage by shuttling electrons from NADH to cytochrome *c* thereby minimizing electron leakage and results in a smaller lesion volume. Additional *in vitro* studied using HT22 cells (neuronal cell line) have demonstrated that MB increases complex I-III activity, cellular oxygen consumption, and glucose uptake [106]. Furthermore, *in vivo* measurements of global glucose uptake, CMRO_2_, global and regional cerebral blood flow were found to be increase with MB treatment [106]. Hypoxic conditions typically result in decreased glucose uptake and decreased cerebral blood flow. MB treatment preserved both glucose uptake and cerebral blood flow compared to normoxic rats when exposed to hypoxic conditions (10% O_2_) [106]. In addition, more recently, our laboratory tested MB’s neuroprotective effects using non-invasive magnetic resonance imaging (MRI) to longitudinally evaluate ischemic evolution in a 60-min transient cerebral ischemia model in rats [107]. Comparisons were made with functional changes using neurological assessments including the 6-point neurological score. The results demonstrated that MB significantly reduces infarct size and behavioral deficits in this experimental model. Moreover, MB markedly salvaged more initial core and penumbral tissues in the cortical regions of the lesion than the vehicle control [107]. Together, these data justify the further exploration of the use of MB as an intervention in early-stages of stroke as it has been shown to inhibit excessive ROS production and maintain mitochondrial function during stress. 

Interestingly, MB has differential effects depending on the dose utilized, with low doses of MB (~1 mg/kg) providing improved conditions in certain disorders while high doses (5–10 mg/kg) cause a worsening of conditions. A prime example of this is the effect of MB on memory enhancement. Low doses of MB have been shown to enhance memory, while high doses have been demonstrated to cause memory impairment [93,100]. An excellent review of the hormetic effects of MB is provided by Rojas *et al.* [93].

## 7. Alternatives to Endogenous MnSOD: MnSOD Mimetics

**Figure 6:**
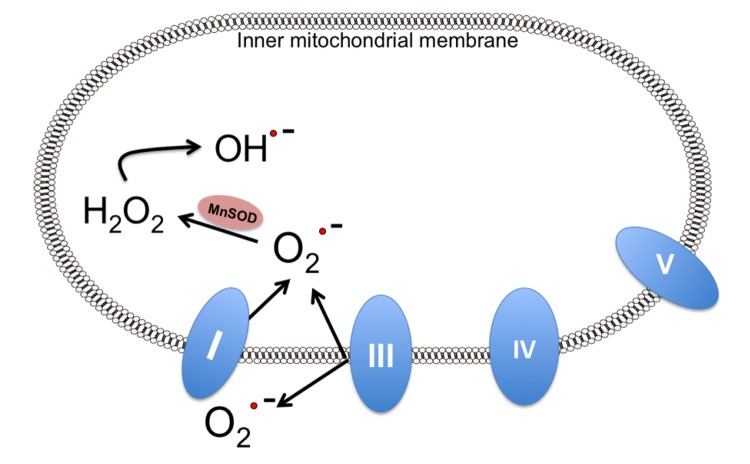
SOD2 plays a critical role in mitochondrial ROS homeostasis. The production of ROS from primarily complex I and III in the oxidative phosphorylation pathway is tightly regulated through antioxidant enzymes with SOD2 playing a major role within the mitochondria converting superoxide radicals to hydrogen peroxide, a less reactive oxygen species.

One vital hallmark of stroke is the production of ROS as described in an earlier section. At basal levels, these species play crucial roles in cell adhesion, growth and differentiation, and in immune responses [108]. This delicate balance between ROS production and their scavenging is maintained by several known mechanisms. Among these are the actions of antioxidant enzymes including superoxide dismutase. Superoxide dismutases (SOD) are a class of enzymes that catalyze the conversion of superoxide to oxygen and hydrogen peroxide and are considered a primary antioxidant defense in cells exposed to ROS. There are three forms of superoxide dismutases: copper-zinc SOD (SOD1, located in the cytoplasm), manganese SOD (SOD2, located in the mitochondria), and SOD3 (located extracellularly). SOD2, specifically, plays a crucial role in mitochondrial ROS homeostasis. Located directly at the site of mitochondrial ROS production, SOD2 catalyzes the breakdown of the superoxide radical in the mitochondrial matrix [109]. However, under the pathological conditions of stroke, markedly increased rates of ROS production lead to a host of issues, such as damage to proteins, lipids, and DNA [110]. Superoxide production is linked to several enzymes within the electron transport chain. NADH ubiquinone oxireductase (complex I), and ubiquinol-cytochrome *c* oxireductase (complex III) produce a disproportionate amount of superoxide radicals (Figure 6). A detailed review of mitochondrial ROS production can be found in a review written by Holley *et al.* [109]. 

Generation of reactive oxidative species (ROS) is an inevitable result of aerobic cellular respiration. While low levels of ROS plays a vital role in processes such as cell adhesion, growth, apoptosis, and immune response, abnormally high levels present during pathological conditions cause damage to vital cellular components [111]. Several endogenous antioxidants, specifically superoxide dismutases (SODs), serve to catalyze the dismutation of superoxide, a radical responsible for damage caused by ischemic oxidative bursts [112]. Several SOD enzymes have been implicated in the reduction of injuries caused by ischemia. Within the CNS, SOD2 is believed to play a major role in counteracting ROS mediated injuries following cerebral ischemia. In addition, deficiencies and overexpression of SOD enzymes give credence to their important role in neuroprotection. For example, SOD2 deficiencies have previously been linked to the exacerbation of cerebral infarcts, whereas overexpression of SOD2 in transgenic mice proved to be neuroprotective [113]. 

There are, however, several properties of the enzyme SOD2 that limit its therapeutic use. Low oral activity, solution instability, brief half-life *in vivo*, and a high molecular weight make the use of SOD2 as a therapeautic agent difficult. Furthermore, oxidation modifications, such as nitration of SOD2 by peryoxynitrate, reduce the concentration of functional enzyme within the mitochondria. Therefore, the efficacy of compounds that mimic SOD activity (SOD mimetics) are currently under evaluation. SOD mimetics possess several qualities that allow for effective use as a therapeutic alternative to SOD. First, they are low molecular weight nonpeptides, which allows for predictable pharmacokinetics and relative ease of diffusion and cell permeability. The molecules are by nature nonimmunogenic, unlike non-human derived SOD. Furthermore, they scavenge superoxide anions with an efficiency similar to that of SOD, and are not deactivated (nitrated) by the toxic anion, peroxynitrate [114]. 

There are three main classes of SOD mimetics, Mn(III) porphyrins, Mn(II) pentaazomacrocyclic and Mn(III) salen complexes [115]. The metal complexes in these molecules mimic the active site of endogenous antioxidant enzymes, and the redox potential of magnesium plays a vital role in their relative activities [116]. Mn(III) porphyrins are composed of four modified pyrrole subunits, which form a heterocyclic macrocycle surrounding a magnesium ion [113] By varying the R groups attached to the porphyrins, both positively and negatively charged species can be synthesized [117]. Because the SOD activity of negatively charged Mn(III) porphyrins is still debated, only positively charged porphyrins are discussed in this review [115]. The neuroprotective effects of one such porphyrin, manganese(III) tetrakis(1-methyl-4-pyridyl)porphyrin (MnTm4PyP) has been recently tested using both *in vivo* and *in vitro* methods. MnTm4Pyp, specifically, exhibited dose dependent reduction of superoxide radical, cytochrome *c*, and cleaved caspase-3 in MCAO mice [112]. Furthermore, *in vitro* analysis of primary cortical neurons exposed to hydrogen peroxide and SOD2 mimetics revealed reduced levels of superoxide and the maintenance of basal intracellular cytosolic Ca^2+^ levels, as compared to controls [117]. The second class of SOD mimetics, Mn(II) pentaazomacrocyclics, have also shown extensive stability and neuroprotection *in vivo*. Experiments assessing the pentaazomacrocyclic, M40403’s efficacy as an antioxidant have shown that it is highly selective for the precursor superoxide radical and does not readily react with other ROS such as hydrogen peroxide, peroxynitrite, and hypochlorous acid. Experiments have shown, however, that the uptake of M40403 into energized mitochondria is negligible. MitoSOD, created by attaching a TPP lipophilic cation to M40403, dramatically improved membrane potential driven uptake of the antioxidant. MitoSOD also exhibited partial protection from intramitochondrial aconitase inactivation by a redox cycler, paraquat. M40403 alone showed no protection, which is possible further proof of differences in mitochondrial uptake. Ultimately, the rate constant of MitoSOD mediated catalysis of superoxide proved smaller than that of endogenous MnSOD. However, because of the high level of accumulation of MitoSOD in the mitochondria, the overall superoxide defense of MitoSOD surpasses that of endogenous SOD [118]. The final SOD mimetics discussed belong to the Mn(III) salen class. Two representatives of the salen complexes, EUK-8 and EUK-134, have shown some promise in reducing both oxidative and nitrosative stresses. Though previously called into question, a recent study has validated the SOD mimetic capabilities of this mimetic class. The same study has also shown that EUK-113, another salen representative, has more than twice the SOD activity of drugs of the same class [115]. An extensive comparison of other SOD mimetics was also conducted [115].

## 8. Conclusion

Current stroke treatments seek to maximize the recovery of neural tissue in proximity to the nutrient deficient core region. It is fairly well established that the maintenance of energy production has a positive impact on reducing damage caused by a stroke and will likely benefit functional recovery. Mitochondria are essential organelles in cell survival, thus, are an important target for therapy. Cells in the penumbra degrade in a time dependent manner when deprived of crucial oxygen and nutrients. For this reason, up-regulation of ATP synthesis by 2meSADP, MRS2365 or methylene blue treatment could play a crucial role in salvaging penumbral tissue. MB’s widely accepted use and ability to reduce the generation of damaging free radicals by decreasing the activity of the aforementioned radical producing complexes in the mitochondria make it a prime candidate for neuroprotection. Purinergic stimulation and methylene blue treatments may offer novel therapeutic regimens in combination or alone to improve patient care following a stroke. In addition, the use of SOD mimetics has also shown promise in maintaining basal cytosolic calcium levels and catalyzing the breakdown of superoxide. Further experimental studies will be needed to determine the significant impact of each of these treatments in both animal and clinical models of stroke. Future study will aim at using multimodal MRI techniques [119,120,121,122,123,124,125,126,127,128] and image analysis techniques [129,130,131] to characterize ischemic tissue (*i.e.*, salvageable versus non- salvageable tissue), evaluate these neuroprotective drugs on different ischemic tissue types, and optimize dosing and treatment time window in a longitudinal fashion.
